# VSL#3^®^ May Reduce Abdominal Pain and Bloating in Ulcerative Colitis Remission with IBS-like Symptoms: An Exploratory Randomized, Double-Blind Placebo-Controlled Trial

**DOI:** 10.3390/nu18142257

**Published:** 2026-07-10

**Authors:** Natalia Borruel Sainz, Xavier Serra-Ruiz, Francisco Guarner Aguilar, Fabiana Castiglione, Olga Maria Nardone, Lucrezia Laterza, Fabio Cascella, Antonio Gasbarrini, Agnieszka Dobrowolska, Alina Kanikowska, Pal Miheller, Orsolya Menyhárt, Daniele Noviello, Flavio Caprioli

**Affiliations:** 1 Crohn’s and Colitis Attention Unit, Gastroenterology Department, Hospital Universitari Vall d’Hebron, 08035 Barcelona, Spain; natalia.borruel@vallhebron.cat (N.B.S.); xavier.serra@vallhebron.cat (X.S.-R.); 2 Department of Clinical Medicine and Surgery, “Federico II” University of Naples, 80131 Naples, Italy; fabcasti@unina.it; 3 Department of Public Health, “Federico II” University of Naples, 80131 Naples, Italy; olga.nardone@libero.it; 4 IBD Unit, Centro di Malattie dell’Apparato Digerente (CeMAD), UOC Medicina Interna e Gastroenterologia, Fondazione Policlinico Universitario “A. Gemelli” IRCCS, 00168 Rome, Italy; lucrezia.laterza@policlinicogemelli.it (L.L.); fabio.cascella01@icatt.it (F.C.); antonio.gasbarrini@policlinicogemelli.it (A.G.); 5 Department of Gastroenterology, Dietetics and Internal Diseases, Poznan University of Medical Sciences, ul. Przybyszewskiego 49, 60-355 Poznan, Poland; agdob@ump.edu.pl (A.D.); akanikowska@ump.edu.pl (A.K.); 6 Institute of Surgery, Transplantation and Gastroenterology, Semmelweis University, Üllői u 78, 1082 Budapest, Hungary; pmiheller@gmail.com (P.M.); orsolya.menyhart@gmail.com (O.M.); 7 Gastroenterology and Endoscopy Unit, Fondazione IRCCS Ca’ Granda Ospedale Maggiore di Milano Policlinico, Department of Pathophysiology and Transplantation, University of Milan, 20122 Milano, Italy; noviellodaniele@gmail.com (D.N.); flavio.caprioli@unimi.it (F.C.)

**Keywords:** inflammatory bowel disease, irritable bowel syndrome, probiotics

## Abstract

**Background:** Irritable bowel syndrome (IBS)-like symptoms are common in patients with ulcerative colitis (UC) in sustained clinical and endoscopic remission and are associated with impaired quality of life. Evidence for targeted treatments in this setting remains limited. **Objective:** To evaluate the efficacy of the multistrain probiotic VSL#3^®^ in patients with UC in stable remission and IBS-like symptoms. **Methods:** In this randomized, double-blind, explorative, placebo-controlled trial, adults with UC in stable remission for ≥6 months and Rome IV C1 or C4 symptoms received VSL#3^®^ 450 billion colony-forming units or placebo twice daily for 8 weeks. The primary endpoint was symptom relief at week 8 assessed by the 5-point Subject’s Global Assessment of Relief scale. Secondary endpoints included Irritable Bowel Syndrome Symptom Severity Scale scores (IBS-SSS), bowel habits, biochemical remission assessed through fecal calprotectin, IBS-QoL, and IBDQ. **Results:** Fifty-five patients were randomized. The primary endpoint did not differ significantly between groups. Between-group differences in IBS-SSS total score were not statistically significant at week 8; however, numerically greater and directionally consistent reductions in symptom severity and abdominal pain were observed with VSL#3^®^ across visits, persisting at follow-up. In exploratory subgroup analyses, restricted to patients with higher abdominal pain and bloating scores at baseline, significant intragroup improvements in abdominal pain and bloating were observed with VSL#3^®^ but not with placebo. Quality of life improvements were numerically greater and more sustained with VSL#3^®^. **Conclusions:** Within the limitations of the small sample size, these results show a signal of benefit in the quality of life of UC patients with IBS-like symptoms and suggest a potential complementary role for VSL#3^®^ in symptom-oriented management, particularly for abdominal pain and bloating. Larger, adequately powered randomized trials are warranted to confirm these exploratory findings.

## 1. Introduction

Up to 30% of ulcerative colitis (UC) patients may refer irritable bowel syndrome (IBS)-like symptoms during periods of clinical and endoscopic remission [1]; more generally, among patients with inflammatory bowel disease (IBD), the prevalence of IBS-like symptoms (assessed using Rome II, Rome III, and Rome IV criteria) has been reported to be approximately 35%, with a higher prevalence in patients with Crohn’s disease (CD) compared with UC (34.9% vs. 29.1%) [2]. These IBS-like symptoms are consistently associated with impaired quality of life [3], commonly evaluated using disease-specific and symptom-based instruments such as the Inflammatory Bowel Disease Questionnaire (IBDQ-32) and the irritable bowel syndrome severity scoring system (IBS-SSS) and do not reflect low-grade inflammatory activity. Patients typically report abdominal bloating or distention, flatulence, constipation, and recurrent diarrhea episodes [4]. Importantly, the symptom profile observed in this population fulfills Rome IV diagnostic criteria for functional bowel disorders, including irritable bowel syndrome and functional abdominal bloating/distension (C1–C4) [5]. Currently, there are no therapeutic interventions with established efficacy specifically targeting IBS-like symptom patients with ulcerative colitis in remission. Evidence in this clinical setting remains limited, and most available data is derived from studies conducted in patients with primary IBS or from other IBD populations. In this regard, a prospective study conducted in patients with quiescent Crohn’s disease reported an improvement in IBS-like symptoms following short-term probiotic supplementation with Bifidobacterium bifidum orally three times a day for 4 weeks [6]. Probiotics, defined as live microorganisms that, when administered in adequate amounts, confer a health benefit to the host [7], have been widely investigated in the management of IBS and functional gastrointestinal disorders. Recent systematic reviews and meta-analyses, including trial sequential, three-level, and network meta-analytic approaches, have suggested that probiotic supplementation may improve global IBS symptoms compared with placebo, although the evidence remains heterogeneous and strain-specific effects remain difficult to establish [8,9,10]. However, data specifically addressing the efficacy of probiotics in patients with ulcerative colitis in remission and concomitant IBS-like symptoms remain scarce. However, data specifically addressing the efficacy of probiotics in patients with ulcerative colitis in remission and concomitant IBS-like symptoms remain scarce. Alterations in gut microbiota composition and in gut–brain axis signaling have been proposed as potential mechanisms contributing to the development and persistence of functional gastrointestinal symptoms in patients with quiescent inflammatory bowel disease [11]. Within this framework, probiotic supplementation represents a plausible therapeutic strategy for targeting IBS-like symptoms in patients with ulcerative colitis in remission. VSL#3^®^ is a highly concentrated multistrain probiotic formulation with a trophic effect on gut paracellular permeability [12]. The product is commercially available in several countries worldwide, including the United States, where it is marketed as a medical food for the dietary management of patients with ulcerative colitis and irritable bowel syndrome. On this basis, we conducted a randomized, double-blind, placebo-controlled, exploratory clinical trial to evaluate the effects of VSL#3^®^ on symptom relief in patients with UC in stable clinical and endoscopic remission and IBS-like symptoms. Additional assessments included IBS symptom severity, UC activity, fecal calprotectin, and quality of life.

## 2. Materials and Methods

### 2.1. Study Design

This was a multicenter, double-blind, randomized, placebo-controlled, parallel-group clinical trial conducted in five European centers (Vall d’Hebron Barcelona Hospital Campus, Fondazione IRCCS Ca’ Granda, Ospedale Maggiore Policlinico di Milano, University Hospital Federico II—Naples, Poznan University of Medical Sciences, Semmelweis University Budapest). All participating hospitals were referral IBD centers with experience in the clinical management of ulcerative colitis and in the conduct of clinical studies in IBD. The study evaluated the efficacy and safety of a high-potency multistrain probiotic formulation (VSL#3^®^) in patients with ulcerative colitis (UC) in stable clinical and endoscopic remission who reported IBS-like functional abdominal symptoms. After a screening period of up to 2 weeks, eligible patients were randomized in a 1:1 ratio to receive either VSL#3^®^ or matching placebo for an 8-week treatment period, followed by a 2-week post-treatment follow-up. Study visits were scheduled at screening (week 2), baseline/randomization (week 0), during treatment (weeks 4 and 8), and at follow-up (week 10). Randomization was performed using a simple randomization list generated by an independent statistician using a validated SAS^®^ program (SAS^®^ for Windows, version 9.4, 64-bit). The randomization list was implemented through the electronic case report form (eCRF) system (Clinical.NET version 4.0). Investigators, study staff, and participants were blinded to treatment allocation throughout the study. The study was conducted in accordance with the principles of the Declaration of Helsinki and Good Clinical Practice guidelines. The protocol was approved by the local Independent Ethics Committees at each participating center, and all patients provided written informed consent prior to any study-related procedures.

### 2.2. Population

#### 2.2.1. Inclusion Criteria

Patients were eligible for inclusion if they met all of the following criteria: age between 18 and 70 years; a previous diagnosis of ulcerative colitis confirmed by endoscopy and histology, including all disease phenotypes (proctitis, left-sided colitis and pancolitis); stable clinical remission for at least 6 months prior to screening, defined by absence of rectal bleeding and no changes in UC-related treatment; stable maintenance therapy with mesalamine, immunosuppressants or biologicals for at least 6 months before inclusion. Patients were required to have evidence of mucosal healing, documented by colonoscopy or rectosigmoidoscopy performed within 12 months prior to the screening visit showing a Mayo endoscopic subscore of 0 or 1. If no endoscopic assessment had been performed within this timeframe, a rectosigmoidoscopy could be carried out during the screening phase to confirm mucosal healing. Eligible patients also had to report IBS-like symptoms according to Rome IV criteria, specifically fulfilling criteria for irritable bowel syndrome (C1) or functional abdominal bloating/distension (C4). Briefly, Rome IV C1 irritable bowel syndrome was defined as recurrent abdominal pain, on average, at least 1 day per week in the last 3 months, associated with two or more of the following: relation to defecation, change in stool frequency, or change in stool form, with symptom onset at least 6 months before diagnosis. Rome IV C4 functional abdominal bloating/distension was defined as recurrent bloating and/or distension occurring, on average, at least 1 day per week, predominating over other symptoms, with insufficient criteria for irritable bowel syndrome and symptom onset at least 6 months before diagnosis [13]. In addition, patients had to be capable of complying with the study protocol and to provide written informed consent prior to participation.

#### 2.2.2. Exclusion Criteria

Patients were excluded from the study if they met any of the following criteria: evidence of clinically active ulcerative colitis at baseline, defined by the presence of rectal bleeding; markedly elevated fecal calprotectin levels at screening (e.g., >500 μg/g); known intolerance to lactose, fructose, or sorbitol; pregnancy and lactation; presence of unstable or clinically significant psychiatric conditions; history of major abdominal surgeries, with the exception of appendectomy. Additional exclusion criteria included inability to comply with the study procedures, and use of probiotics or topical and/or systemic antibiotic therapy within the 2 months preceding screening.

#### 2.2.3. Treatment Plan

Eligible patients were instructed to maintain their usual dietary habits throughout the entire duration of the study. At baseline (week 0), patients were randomized in a 1:1 ratio to receive either the active treatment or placebo. The active treatment consisted of a high-potency multistrain probiotic formulation (VSL#3^®^ Actial Farmaceutica Srl, Rome, Italy; Lots G1C046, G2B048, and G3F180, manufactured by Biofarma Srl, Gallarate, Italy on 03/2021, 02/2022, and 06/2024, respectively), administered as one sachet containing 450 billion colony-forming units, taken orally twice daily (during breakfast and dinner) for 8 weeks. The probiotic formulation comprised eight bacterial strains: *Streptococcus thermophilus* BT01, *Bifidobacterium breve* BB02, *Bifidobacterium animalis* subsp. *lactis* BL03, *Bifidobacterium animalis* subsp. *lactis* BI04, *Lactobacillus acidophilus* BA05, *Lactobacillus plantarum* BP06, *Lactobacillus paracasei* BP07, and *Lactobacillus helveticus* BD08. Excipients included maltose, cornstarch, and silicon dioxide. Patients randomized to the control group received a matching placebo containing only excipients, provided in identically packaged sachets. The placebo was indistinguishable from the active product in appearance, smell, and taste. Study treatment was dispensed at baseline, and treatment compliance was assessed at each study visit. Concomitant maintenance therapies for ulcerative colitis remained unchanged throughout the study period, whereas the use of probiotics and topical or systemic antibiotics was prohibited during the trial.

### 2.3. Study Objectives and Endpoints

The primary objective of the study was to evaluate the effect of VSL#3^®^ on self-reported severity of functional abdominal symptoms in patients with ulcerative colitis in stable clinical remission and IBS-like symptoms. The primary endpoint was the self-assessment of relief of symptoms at week 8 of treatment, assessed using the validated Subject’s Global Assessment (SGA) of Relief 5-point scale [14]. The SGA of Relief scale is a patient-reported assessment of overall symptom relief and includes the following response options: completely relieved, considerably relieved, somewhat relieved, unchanged, and worse. Patients were classified as responders if they reported being “completely relieved” or “considerably relieved”. The primary analysis was based on the comparison of responder rates between treatment groups. Secondary endpoints were to evaluate the difference between the two groups in the responder rates at week 4; in the composite and individual symptom scores of the validated quantitative Irritable Bowel Syndrome Symptom Severity Scale (IBS-SSS) [15] at weeks 4, 8, and 10; in the Simple Clinical Colitis Activity Index (SCCAI) [16] at weeks 4, 8 and 10; in fecal calprotectin at weeks 4 and 8; in the Irritable Bowel Syndrome Quality of Life (IBS QoL) index, a validated disease-specific instrument for assessing quality of life in patients with irritable bowel syndrome at weeks 4, 8 and 10 [17]; and in the Inflammatory Bowel Disease Questionnaire (IBDQ) index, a validated disease-specific quality of life questionnaire for patients with inflammatory bowel disease, at week 4, 8 and 10 [18].

### 2.4. Statistical Analysis

Efficacy analyses were performed on the Full Analysis Set (FAS) population, defined as all randomized patients who received at least one dose of study treatment and had at least one post-baseline assessment of the primary endpoint. A per-protocol (PP) population, including patients without major protocol deviations, was used for supportive analyses of primary and secondary endpoints. Safety analyses were conducted in the safety population, comprising all patients who received at least one dose of study treatment. Descriptive statistics were used to summarize baseline characteristics and study outcomes, including frequencies and percentages for categorical variables and means and standard deviations of continuous variables. The primary endpoint was analyzed by comparing responder rates at week 8 between treatment groups using the Chi square test. Analyses were performed both on available Week 8 Subject’s Global Assessment (SGA) of Relief data and using a conservative non-responder imputation approach, whereby subjects without a week-8 SGA assessment were classified as non-responders. *t*-tests were used to compare the change from baseline of IBS-SSS total score and individual items between the two groups. To further explore changes over time in IBS symptom severity, a mixed linear regression model was implemented considering IBS-SSS total score as the dependent variable, patients and center as random factors and baseline values, week and interaction between treatment and week as fixed factors. In the subgroup patients symptomatic for pain at baseline (excluding patients with baseline score of 0 for IBS-SSS pain items) a Wilcoxon test was performed to evaluate the change from baseline in each group separately and the Mann–Whitney test was used to compare changes between groups. Chi square was used to compare the proportion of patients achieving a clinically meaningful reduction in abdominal pain (≥30% reduction from baseline, according to FDA recommendations [19] between the two groups). The mixed logistic regression model on relapse in ulcerative colitis activity based on the SCCAI scale was implemented including treatment group as the fixed factor and center as the random factor. Odds ratio and its relative confidence at 95% were reported. Relapse was defined as an SCCAI total score ≥ 5. Mixed linear models were implemented with IBS-QoL total score and IBDQ as the dependent variables, patients and center as random factors and baseline values, week and interaction between treatment and week as fixed factors. Estimated means for each time point and change from baseline were reported with difference in reduction between the two groups and the relative CI95%. For all mixed-effects model analyses, all available post-baseline observations were included in the estimation of model-based marginal means. Missing post-baseline data were handled within the model estimation procedure, without explicit imputation. To evaluate change in a single item of IBDQ, the Wilcoxon test was performed to evaluate the change from baseline in each group separately and the Mann–Whitney test was used to compare changes between groups. Secondary categorical endpoints, including responder rates at week 4, were analyzed using the same modeling approach. The values of fecal calprotectin ≤50 were set =50; the Wilcoxon test was performed to evaluate the change from baseline. All statistical tests were two-sided, and a *p*-value < 0.05 was considered statistically significant. No adjustment for multiple comparisons was performed for secondary endpoints. All analyses were conducted using SAS^®^ software (version 9.4).

### 2.5. Sample Size Calculation

The sample size was estimated based on the primary endpoint (responder rate at week 8 on the SGA of Relief scale). The study assumed a responder rate of 65% in the VSL#3^®^ group and 40% in the placebo group. Using a two-sided significance level (α) of 0.05 and a type II error rate (β) of 0.20 (power 80%), 62 patients per treatment arm were required, for a planned total sample size of 124 patients.

## 3. Results

**Figure 1:**
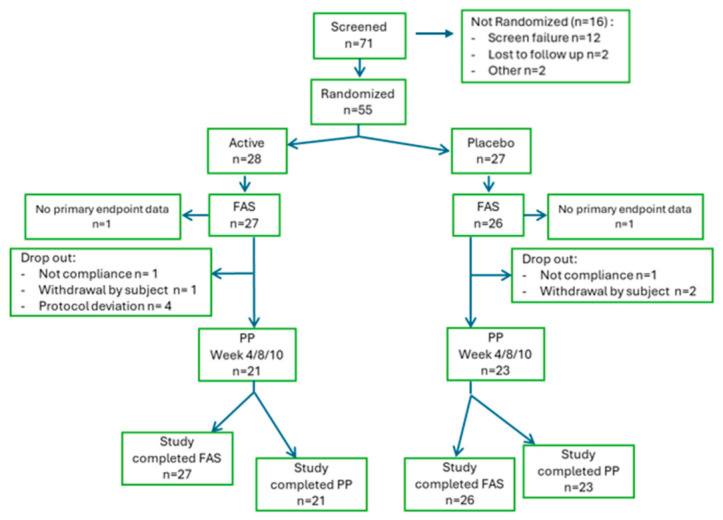
Flow of participants and composition of analysis populations. Flow diagram of patient disposition throughout the study, including the number of subjects screened, reasons for screening failure, randomized patients, and allocation to treatment groups. The figure also reports the composition of the Full Analysis Set (FAS), defined as all randomized patients who received at least one dose of study treatment and had at least one post-baseline assessment, and the per-protocol (PP) population, defined according to predefined protocol criteria. Numbers are presented as absolute counts.

The study was prematurely interrupted by the Sponsor due to slow recruitment rate. Therefore, efficacy analysis was considered as exploratory. Overall, 55 patients were randomized compared with the planned sample size of 124. Figure 1 resumes reason for screening failure and the composition of study FAS and PP population. Patients’ characteristics at baseline are reported in Table 1.

No differences were observed between VSL#3 and placebo groups in the primary endpoint at week 8, defined as the proportion of responders according to the Subject’s Global Assessment of Relief (Table 2).

Similarly, no statistically significant differences were observed between treatment groups in the mean IBS-SSS total score or individual item scores at week 8 (Table 3). Across visits, patient treated with VSL#3 showed numerically greater reductions from baseline in the IBS-SSS total score and in the abdominal pain severity item, with effects already evident at week 4, maintained at the end of treatment, and persisting during follow-up.

**Figure 2:**
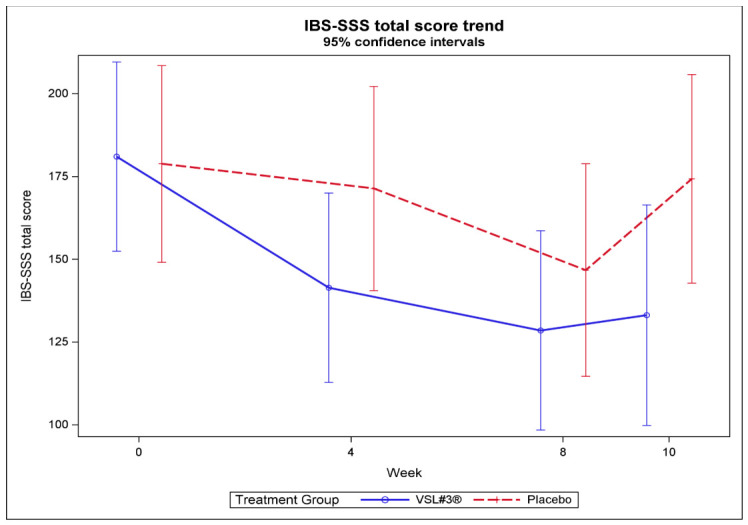
Change from baseline in IBS-SSS total score over time (Full Analysis Set). Estimated mean change from baseline in Irritable Bowel Syndrome Symptom Severity Scale (IBS-SSS) total score at weeks 4, 8, and 10 in the Full Analysis Set (FAS). Results are derived from a mixed-effects model for repeated measures including treatment group, visit, treatment-by-visit interaction, study site, baseline value, and the relevant interaction terms, as specified in the statistical analysis plan. Data are presented as model-based estimates with 95% confidence intervals. Symbols represent treatment groups.

Results from the mixed model regression showed numerically greater reductions from baseline in IBS-SSS total score in patients treated with VSL#3^®^ compared with placebo at weeks 4, 8 and 10. Although between-group differences did not reach statistical significance, the direction and magnitude of the estimated effects were consistent over time (Figure 2).

Exploratory analyses focusing on patients symptomatic for pain at baseline (excluding patients with baseline score of 0 for IBS-SSS pain items) showed a reduction from baseline in abdominal pain severity with statistically significant intragroup improvement observed at week 8 and pain frequency (number of days with pain over 10 days) over time in the VSL#3^®^ group, with statistically significant intragroup improvement observed at weeks 4, 8 and 10, while no significant intragroup changes were observed in the placebo group (Table 4).

**Figure 3:**
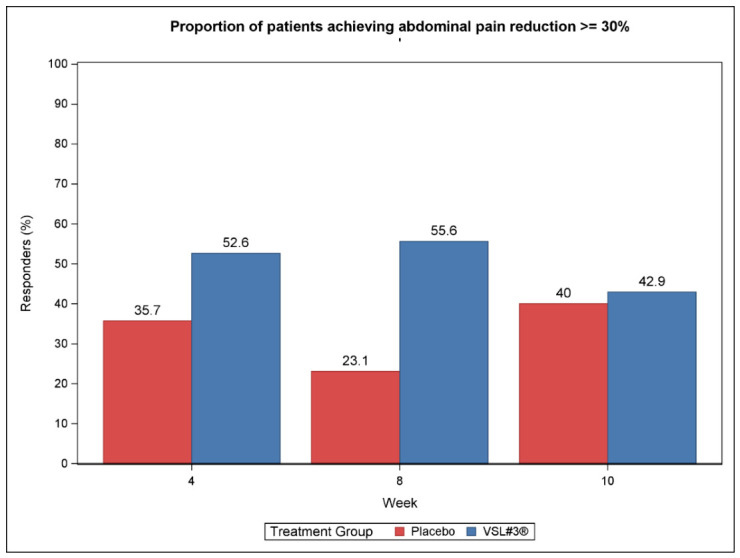
Proportion of patients achieving ≥30% reduction in abdominal pain from baseline (Full Analysis Set). Proportion of patients in the Full Analysis Set (FAS) with a baseline abdominal pain score different from 0 who achieved a ≥30% reduction from baseline at weeks 4, 8, and 10. The ≥30% threshold was defined in accordance with FDA recommendations for clinically meaningful improvement in abdominal pain. Data are presented as n (%). Between-group comparisons were performed using a logistic regression model including treatment group, study site, treatment-by-site interaction, and baseline value as covariates.

In a complementary responder analysis, the proportion of patients with a baseline value other than 0 achieving a clinically meaningful reduction in abdominal pain (≥30% decrease from baseline, in accordance with FDA recommendations) was numerically higher in the VSL#3^®^ group compared with placebo; however, the between-group difference did not reach statistical significance (Figure 3).

**Figure 4:**
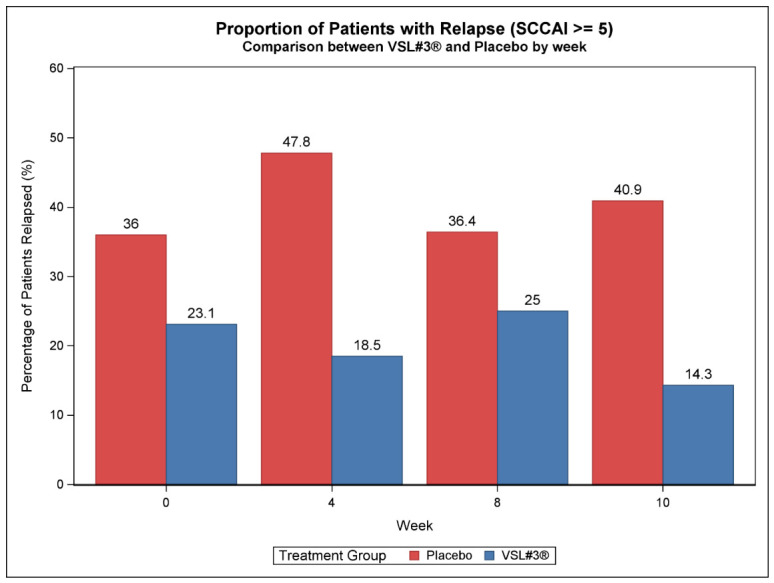
Proportion of patients with relapse (SCCAI ≥ 5) during follow-up (Full Analysis Set). Proportion of patients in the Full Analysis Set (FAS) meeting criteria for relapse at weeks 4, 8, and 10, defined as a Simple Clinical Colitis Activity Index (SCCAI) total score ≥ 5. Data are presented as n (%). Between-group comparisons were performed using a logistic regression model including treatment group, study site, treatment-by-site interaction, and baseline value as covariates.

Ulcerative colitis activity was assessed at each follow-up visit using the Simple Clinical Colitis Activity Index (SCCAI). Across follow-up visits, the proportion of patients experiencing relapse (defined as SCCAI total score ≥ 5) was numerically lower in the VSL#3^®^ group compared with placebo (Figure 4).

A logistic regression model was applied to estimate the odds of relapse at each visit. At week 4, treatment with VSL#3^®^ was associated with an approximately 80% reduction in the odds of relapse compared with placebo (OR 0.20; 95% CI: 0.05–0.81; *p* = 0.0249). At week 8, no evidence of a treatment effect was observed (OR 0.59; 95% CI: 0.15–2.34; *p* = 0.4470). At week 10 the estimated odds ratio again favored VSL#3^®^ (OR 0.23; 95% CI: 0.05–1.09); however, this difference did not reach statistical significance (*p* = 0.0636).

Analysis of fecal calprotectin μg/g revealed stable inflammation levels during the study, with no clinically relevant variations in either group (Supplementary Table S1).

IBS-specific quality of life was assessed using the IBS-QoL questionnaire at weeks 4, 8, and 10. In the mixed model analysis, a significant increment in IBS-QOL for the VSL#3^®^ group at each week (only at week 8 in the placebo group) was found. The changes from baseline are higher in the VSL#3^®^ group than in the placebo group at each week and reach a statistically significant difference at week 10 (Table 5).

When IBS-QoL individual domains were explored, patients treated with VSL#3^®^ showed numerically greater and more sustained improvements across selected functional domains compared with placebo. In particular, between-group differences favoring VSL#3^®^ were observed for Interference with Activity at weeks 4 and 10, and for Health Worry and Relationship at week 10. Directionally consistent improvements were also observed in Food Avoidance and Social Reaction, with changes persisting through follow-up. Detailed domain-level results are reported in Supplementary Table S2.

In within-group analyses based on a mixed model, in the Inflammatory Disease Questionnaire (IBDQ), both treatment groups showed a statistically significant improvement from baseline at week 8, with mean changes of +8.9 points in the VSL#3^®^ group (*p* = 0.0165) and +8.5 points in the placebo group (*p* = 0.0272). At week 10, a statistically significant improvement from baseline was maintained in the VSL#3^®^ group (+12.8 points; *p* = 0.0011), whereas the change from baseline in the placebo group was no longer statistically significant (+5.4 points; *p* = 0.1571) (Supplementary Table S3).

Furthermore, abdominal bloating was evaluated using the specific IBDQ item and restricting the analysis to patients with clinically relevant bloating at baseline, excluding patients who had a baseline score of 7 corresponding to no symptoms (“none of the time”), for the item “How much of the time during the last 2 weeks have you been troubled by a feeling of abdominal bloating?”. In this subgroup, a significant intragroup reduction was observed in the VSL#3^®^ group at weeks 4 and 10, whereas no significant changes were detected in the placebo group (Table 6).

Adverse events were monitored throughout the study period. No clinically relevant differences in the incidence of adverse events were observed between the VSL#3^®^ and placebo groups. No safety signals emerged, and no treatment-related serious adverse events were reported.

## 4. Discussion

In recent years, the proportion of patients with IBD in a quiescent phase has increased as a result of advanced therapies. Despite sustained clinical and endoscopic remission, IBS-like symptoms remain common in patients with ulcerative colitis (UC). Reported prevalence estimates range from approximately 18–32% in inactive UC, corresponding to a two- to three-fold higher frequency compared with healthy controls [20,21]. Accurate estimation of prevalence is challenged by variability in remission definitions across studies. In this regard, a prospective study by Olsen et al. [3], using objective markers of disease quiescence, reported IBS-like symptoms in 21.9% of patients when remission was defined based on fecal calprotectin levels, decreasing to 19.2% when remission was based on endoscopic assessment. The most frequently reported symptoms include abdominal pain, bloating and altered bowel habits, often associated with psychological comorbidities, such as anxiety and depression, that may further amplify symptom burden [22,23,24]. Management of IBS-like symptoms in patients with quiescent UC is therefore complex and typically requires a multifactorial approach, including dietary interventions such as a low FODMAPs diet [25] and treatments targeting visceral hypersensitivity or motility [26,27] that largely mirror those adopted for general management of IBS [28,29,30]. Probiotic supplementation has also been evaluated in the management of IBS. Recent meta-analyses and network meta-analyses of randomized controlled trials in IBS suggest that probiotics may provide modest improvements in global IBS symptoms compared with placebo, although results are heterogeneous across probiotic strains, formulations, treatment durations, and outcome definitions [8,9,10]. Importantly, these data mainly derive from primary IBS populations rather than from patients with ulcerative colitis in remission and IBS-like symptoms and therefore should be extrapolated cautiously. However, the available evidence is heterogeneous, and data specifically addressing patients with ulcerative colitis in remission and concomitant IBS-like symptoms remain limited.

The present study evaluated the effect of VSL#3^®^ in patients with UC in stable remission presenting with IBS-like symptoms. Because only 55 patients were randomized compared with the planned sample size of 124, the study was underpowered to detect between-group differences, particularly for the primary responder-based endpoint. As a consequence, the absence of a statistically significant difference in the SGA of Relief scale cannot be interpreted as definitive evidence of no treatment effect. Conversely, the numerical trends observed across secondary and exploratory endpoints should not be interpreted as confirmatory, but rather as hypothesis-generating findings requiring confirmation in adequately powered randomized trials. Furthermore, the SGA of Relief scale, although validated and widely used in functional gastrointestinal disorders, is known to be sensitive to placebo response and may be less suited to capture gradual or multidimensional symptom changes in heterogeneous patient populations such as those with IBS-like symptoms during ulcerative colitis remission. Finally, despite microbiota and gut–brain axis alterations providing a plausible rationale for probiotic supplementation in this setting, the present trial did not include microbiome or mechanistic biomarker analyses. Therefore, the study was able to evaluate clinical and patient-reported outcomes, but not to determine whether symptom changes were mediated by modifications in microbiota composition, microbial function, intestinal permeability, or gut–brain signaling.

Abdominal pain represents one of the most relevant and burdensome IBS-like symptoms reported by patients with UC in remission. In the present study, treatment with VSL#3^®^ was associated with numerically greater reductions from baseline in IBS Symptom Severity Scale (IBS-SSS) total score and in the abdominal pain severity item compared with placebo at the end of the treatment. Other studies have evaluated the efficacy of probiotics in reducing pain, and although they were not specifically conducted in patients with UC in remission, the results support the effectiveness of probiotic treatment [31,32]. Although between-group differences did not reach statistical significance, analyses based on a mixed-effects regression model showed a directionally consistent treatment effect favoring VSL#3^®^ across all assessed time points. The stability and persistence of these estimates over time suggest a coherent symptomatic signal. Abdominal bloating and distension are among the most frequently reported and distressing IBS-like symptoms in patients with ulcerative colitis in remission. In the present study, assessment of bloating using the abdominal distension item of the IBS-SSS did not reveal relevant differences over time, either within or between treatment groups. By contrast, when bloating was evaluated using the disease-specific Inflammatory Bowel Disease Questionnaire (IBDQ) and analyses were restricted to patients with clinically relevant bloating at baseline, treatment with VSL#3^®^ was associated with a significant intragroup improvement at weeks 4 and 10, whereas no significant changes were observed in the placebo group. Subgroup analyses restricted to patients with baseline abdominal pain or clinically relevant bloating were exploratory, and should be interpreted as hypothesis-generating rather than confirmatory. Although exploratory in nature, these findings suggest that disease-specific quality of life instruments may be more sensitive in detecting changes in bloating-related symptom burden in patients with ulcerative colitis in remission, particularly when baseline symptom severity is taken into account. Beyond symptom severity, IBS-like functional abdominal complaints have a substantial impact on patients’ daily functioning and perceived well-being. In the present study, improvements in IBS-specific quality of life were more pronounced and sustained over time in patients treated with VSL#3^®^ compared with placebo, suggesting that symptomatic changes may translate into relevant patient-reported benefits. In this context, the improvement in quality of life and symptom severity that had a numerically greater result for VSL#3^®^ should be interpreted as explorative and not conclusive, as it should be confirmed in a adequately powered study. At the domain level, the greater and more sustained improvement in the IBS-QoL total score with VSL#3^®^ appeared to be mainly driven by changes in domains related to daily activity, symptom-related concerns, relationships, food avoidance, and social reaction. This pattern suggests that the observed symptomatic trends may have affected several aspects of daily functioning. When disease-specific quality of life was assessed using the IBDQ, both treatment groups showed an improvement during the treatment phase, consistent with the inclusion of patients in clinical remission. However, only patients receiving VSL#3^®^ maintained this improvement at follow-up, indicating that relief of IBS-like symptoms may contribute to a more durable perception of well-being even in the absence of changes in inflammatory disease activity. UC activity was monitored throughout this study using the Simple Clinical Colitis Activity Index (SCCAI). Although this trial was not powered to detect differences in disease activity outcomes, patients treated with VSL#3^®^ consistently showed numerically lower relapse rates compared with placebo across follow-up visits. These results confirm what has emerged from studies evaluating the efficacy of probiotics in inducing and maintaining remission in patients with ulcerative colitis, such as the study by Li-Xuan Sang et al. [33].

Results from mixed-effects logistic regression analyses indicated a reduced odds of relapse in the VSL#3^®^ group at early time points, while estimates at later visits continued to directionally favor the active treatment without reaching statistical significance. These observations should be interpreted cautiously, given the limited sample size and the exploratory nature of the analyses. Importantly, probiotic supplementation was not associated with worsening of disease activity, and clinical stability was maintained throughout the study period. Interpretation of SCCAI-based outcomes in this context is further complicated by the overlap between functional abdominal symptoms and components of the activity index, which incorporates symptoms that may overlap with functional abdominal complaints. In patients with ulcerative colitis in clinical remission but with IBS-like symptoms, functional complaints such as abdominal pain and altered bowel habits may influence SCCAI scores independently of inflammatory activity. This overlap may partly account for the variability observed in SCCAI values over time and may influence activity scores independently of underlying inflammation and should be taken into account when interpreting SCCAI-based outcomes. Furthermore, the study population included patients with different IBS-like symptom patterns and varying baseline symptom severity. While reflective of real-world clinical practice, this heterogeneity may have diluted treatment effects in the overall analysis, as suggested by the more pronounced and consistent improvements observed in exploratory analyses restricted to patients symptomatic at baseline. In this context, the directional trend toward improvement in functional bloating and abdominal pain observed in the present study is broadly consistent with observations reported in IBS populations, where dietary interventions, probiotic supplementation, and pharmacological approaches have been associated with reductions in bloating-related symptoms [34]. However, differences in patient populations and study designs warrant caution when extrapolating findings from IBS to patients with ulcerative colitis in remission.

## 5. Conclusions

In conclusion, the findings of the present study are best interpreted by considering the overall pattern of results across secondary and exploratory endpoints. Although the primary responder-based endpoint did not differ between treatment groups, patients treated with VSL#3^®^ showed numerically greater improvements compared with placebo across multiple symptom-related measures and time points, particularly for abdominal pain-related outcomes and patient-reported quality of life indices, without evidence of safety concerns. Taken together, these results suggest a possible signal of symptomatic benefit within the limitations imposed by the reduced sample size. Furthermore, these findings support the hypothesis that targeting IBS-like symptoms represents a relevant therapeutic objective in IBS overlap to UC patients in remission and suggests that probiotics may play a complementary role in symptom-oriented management strategies. These findings suggest a possible symptomatic signal but do not allow definitive conclusions regarding efficacy. Larger, adequately powered randomized trials are warranted to confirm these exploratory findings and to better define the role of probiotic interventions in UC patients with persistent IBS-like symptoms during remission.

## Figures and Tables

**Table 1:** Patient characteristics at baseline. Baseline demographic and clinical characteristics of patients included in the Full Analysis Set (FAS), stratified by treatment group. Data are reported as mean (SD) or *n* (%), as appropriate. Percentages are calculated within treatment groups.

	VSL#3^®^ (%, *n*/N)	Placebo (%, *n*/N)	Total (%, *n*/N)
Full Analysis Set, *n*			
	27	26	53
Age (years)			
N	27	26	53
Mean (SD)	46.8 (14.85)	47.2 (12.48)	47.0 (13.61)
Gender, *n* (%)			
Female	55.6% (15/27)	65.4% (17/26)	60.4% (32/53)
Male	44.4% (12/27)	34.6% (9/26)	39.6% (21/53)
IBS-SSS baseline			
0	11.1% (3/27)	24.0% (6/25)	17.3% (9/52)
Mild	29.6% (8/27)	24.0% (6/25)	26.9% (14/52)
Moderate	51.9% (14/27)	44.0% (11/25)	48.1% (25/52)
Severe	7.4% (2/27)	8.0% (2/25)	7.7% (4/52)
Summary SCCAI score baseline			
N	26	22	48
Mean (SD)	3.4 (1.36)	4.1 (2.19)	3.8 (1.80)
SCCAI score baseline			
1	11.5% (3/26)	13.6% (3/22)	12.5% (6/48)
2	11.5% (3/26)	13.6% (3/22)	12.5% (6/48)
3	26.9% (7/26)	13.6% (3/22)	20.8% (10/48)
4	26.9% (7/26)	18.2% (4/22)	22.9% (11/48)
5	19.2% (5/26)	13.6% (3/22)	16.7% (8/48)
6	3.8% (1/26)	4.5% (1/22)	4.2% (2/48)
7	0	18.2% (4/22)	8.3% (4/48)
8	0	4.5% (1/22)	2.1% (1/48)
Disease extension			
Proctitis	29.6% (8/27)	23.1% (6/26)	26.4% (14/53)
Left side colitis	33.3% (9/27)	23.1% (6/26)	28.3% (15/53)
Extensive colitis	37.0% (10/27)	53.8% (14/26)	45.3% (24/53)
Symptoms part of Rome IV criteria			
C1 Irritable bowel syndrome	29.6% (8/27)	15.4% (4/26)	22.6% (12/53)
C4 Functional abdominal bloating/distention	70.4% (19/27)	84.6% (22/26)	77.4% (41/53)
Concomitant medication for UC therapy			
Antidiarrheals, intestinal			
Anti-inflammatory/anti-infective agents			
Mesalazine	78.6% (22/28)	85.2% (23/27)	81.8% (45/55)
Sulfasalazine	3.6% (1/28)	0	1.8% (1/55)
Corticosteroids for systemic use			
Methylprednisolone	3.6% (1/28)	3.7% (1/27)	3.6% (2/55)
Prednisone	3.6% (1/28)	3.7% (1/27)	3.6% (2/55)
Immunosuppressants			
Adalimumab	3.6% (1/28)	18.5% (5/27)	10.9% (6/55)
Azathioprine	3.6% (1/28)	3.7% (1/27)	3.6% (2/55)
Golimumab	0	3.7% (1/27)	1.8% (1/55)
Infliximab	14.3% (4/28)	3.7% (1/27)	9.1% (5/55)
Methotrexate sodium	3.6% (1/28)	0	1.8% (1/55)
Tofacitinib	0	11.1% (3/27)	5.5% (3/55)
Ustekinumab	7.1% (2/28)	0	3.6% (2/55)
Vedolizumab	17.9% (5/28)	7.4% (2/27)	12.7% (7/55)

**Table 2:** Proportion of responder patients based on the Subject’s Global Assessment (SGA) of Relief scale at week 8. Distribution of SGA of Relief scale categories and responder status at week 8 in the Full Analysis Set. Responders were defined as patients reporting as “completely relieved” or “considerably relieved.” Percentages are calculated within treatment groups among patients who completed the visit; analyses with imputation consider patients with missing assessments as non-responders.

	VSL#3^®^ (%, *n*/N)	Placebo (%, *n*/N)	Total (%, *n*/N)	*p*-Value
Full Analysis Set, *n*				
	27	26	53	
Week 8				
Number of patients who performed				
	88.9% (24/27)	84.6% (22/26)	86.8% (46/53)	
Subject’s Global Assessment (SGA) scale				
Completely relieved	0	13.6% (3/22)	6.5% (3/46)	
Considerably relieved	29.2% (7/24)	13.6% (3/22)	21.7% (10/46)	
Somewhat relieved	20.8% (5/24)	31.8% (7/22)	26.1% (12/46)	
Unchanged	37.5% (9/24)	36.4% (8/22)	37.0% (17/46)	
Worse	12.5% (3/24)	4.5% (1/22)	8.7% (4/46)	
Subject’s Global Assessment (SGA) response				
Non-responder	70.8% (17/24)	72.7% (16/22)	71.7% (33/46)	0.8867
Responder	29.2% (7/24)	27.3% (6/22)	28.3% (13/46)	
Subject’s Global Assessment (SGA) response with imputation				
Non-responder	74.1% (20/27)	76.9% (20/26)	75.5% (40/53)	0.8096
Responder	25.9% (7/27)	23.1% (6/26)	24.5% (13/53)	

**Table 3:** Summary of IBS Symptom Severity Scale (IBS-SSS) individual items and total score by week. Mean scores and change from baseline for individual IBS-SSS items and total score at each study visit in the Full Analysis Set. Data are presented by treatment group and visit.

				Change from Baseline			
	N	Mean	SD	N	Mean	SD	*p* Value
How severe is your abdominal (tummy) pain?							
Placebo							
Baseline	25	24.80	26.48				
Week 4	23	22.61	30.33	23	−1.30	27.02	
Week 8	22	25.91	28.89	21	−0.48	24.79	
Week 10	22	21.82	26.84	22	−3.18	25.89	
VSL#3^®^							
Baseline	27	29.26	28.81				
Week 4	27	20.37	27.52	27	−8.89	29.26	0.3459
Week 8	24	19.58	27.10	24	−13.33	30.60	0.1271
Week 10	19	22.11	28.40	19	−12.63	28.45	0.2761
Number of days that you get pain in every 10 days							
Placebo							
Baseline	25	2.56	2.84				
Week 4	23	2.04	3.02	23	−0.43	2.15	
Week 8	22	2.18	2.70	21	−0.52	1.97	
Week 10	22	2.45	3.28	22	−0.14	2.40	
VSL#3^®^							
Baseline	27	3.30	3.51				
Week 4	27	2.11	3.36	27	−1.19	2.97	0.3073
Week 8	24	1.92	3.03	24	−1.79	2.92	0.0918
Week 10	19	1.89	2.81	19	−1.68	2.94	0.0762
How severe is your abdominal distension/tightness?							
Placebo							
Baseline	25	40.40	29.37				
Week 4	23	30.43	31.98	23	−10.00	33.03	
Week 8	22	32.73	31.80	21	−8.57	31.03	
Week 10	22	32.27	31.91	22	−8.18	34.31	
VSL#3^®^							
Baseline	27	35.19	30.18				
Week 4	27	27.78	30.04	27	−7.41	34.26	0.7869
Week 8	24	26.67	25.14	24	−10.83	38.33	0.8280
Week 10	19	26.32	30.95	19	−8.42	35.48	0.9827
How satisfied are you with your bowel habit?							
Placebo							
Baseline	25	44.80	30.43				
Week 4	23	50.43	27.71	23	7.83	35.67	
Week 8	22	37.73	31.91	21	−6.19	37.61	
Week 10	22	49.09	26.89	22	6.36	23.21	
VSL#3^®^							
Baseline	27	48.89	21.00				
Week 4	27	47.04	32.20	27	−1.85	38.33	0.3602
Week 8	24	44.17	31.20	24	−3.75	38.88	0.8318
Week 10	19	43.68	29.48	19	−4.21	31.68	0.2378
How much is your IBS affecting or interfering your life in general?							
Placebo							
Baseline	25	40.00	28.43				
Week 4	23	39.57	29.15	23	1.30	29.89	
Week 8	22	29.09	24.86	21	−10.48	22.47	
Week 10	22	40.45	27.68	22	2.27	21.14	
VSL#3^®^							
Baseline	27	38.52	25.53				
Week 4	27	28.89	32.62	27	−9.63	22.27	0.1561
Week 8	24	27.92	29.04	24	−9.17	32.29	0.8741
Week 10	19	31.58	23.87	19	−5.79	22.44	0.2461
IBSS Total Score							
Placebo							
Baseline	25	175.60	111.13				
Week 4	23	163.48	118.61	23	−6.52	94.75	
Week 8	22	147.27	116.67	21	−30.95	82.52	
Week 10	22	168.18	125.65	22	−4.09	90.01	
VSL#3^®^							
Baseline	27	184.81	97.05				
Week 4	27	145.19	95.41	27	−39.63	72.98	0.1795
Week 8	24	137.50	107.59	24	−55.00	109.90	0.4079
Week 10	19	142.63	124.85	19	−47.89	108.35	0.1718

**Table 4:** Change from baseline in IBS-SSS pain severity and pain frequency in symptomatic patients. Change from baseline in IBS-SSS abdominal pain severity and pain frequency (number of days with pain over 10 days) by visit in the subgroup of patients symptomatic at baseline. Patients with a baseline score of 0 for the respective item were excluded. * *p*-value from non-parametric test. ** *p*-value from non-parametric intragroup test. Data are presented for the Full Analysis Set.

				Change from Baseline				
	N	Mean	SD	N	Mean	SD	*p* Value *	*p* Value **
How severe is your abdominal (tummy) pain?								
Placebo								
Baseline	13	47.69	14.81					
Week 4	12	35.83	30.29	12	−10.00	27.63		0.2782
Week 8	12	38.33	27.91	11	−10.91	22.12		0.3924
Week 10	12	30.00	27.30	12	−15.83	20.65		0.1043
VSL#3^®^								
Baseline	17	46.47	22.34					
Week 4	17	28.24	30.46	17	−18.24	31.87	0.2889	0.0673
Week 8	17	25.29	29.18	17	−21.18	32.19	0.1836	0.0244
Week 10	14	30.00	29.35	14	−17.14	32.21	0.4587	0.0814
Number of days that you get pain in every 10 days								
Placebo								
Baseline	13	4.92	1.89					
Week 4	12	3.50	3.53	12	−1.25	2.60		0.0869
Week 8	12	3.50	2.88	11	−1.55	1.92		0.1541
Week 10	12	3.83	3.71	12	−0.92	2.71		0.3243
VSL#3^®^								
Baseline	17	5.24	3.03					
Week 4	17	2.94	3.85	17	−2.29	3.06	0.1804	0.0206
Week 8	17	2.65	3.35	17	−2.59	3.14	0.3259	0.0109
Week 10	14	2.57	3.01	14	−2.29	3.24	0.2183	0.0125

**Table 5:** Mixed model and marginal means for IBS Quality of Life (IBS-QoL) total score. Results of the mixed-effects model evaluating changes from baseline in IBS-QoL total score over time in the Full Analysis Set. The model includes patient and study center as random effects and baseline value, visit, treatment group, and treatment-by-visit interaction as fixed effects. Marginal means and descriptive statistics are reported.

			Change from Baseline				Difference in Reduction		
	VSL#3^®^	Placebo	VSL#3^®^	*p* Value	Placebo	*p* Value	Mean (se)	CI 95%	*p* Value
Baseline	66.42 (1.28)	66.26 (1.29)							
Week 4	70.26 (1.68)	69.18 (1.78)	3.85 (1.93)	0.0484	2.92 (2.02)	0.1503	−0.93 (2.80)	(−6.45; 4.60)	0.7412
Week 8	72.45 (1.78)	71.32 (1.86)	6.03 (2.02)	0.0033	5.06 (2.09)	0.0166	−0.97 (2.91)	(−6.72; 4.77)	0.7381
Week 10	72.07 (1.95)	65.43 (1.82)	5.66 (2.18)	0.0102	−0.83 (2.06)	0.6861	−6.49 (3.00)	(−12.4; −0.58)	0.0317

**Table 6:** Change from baseline in the IBDQ bloating item. Change from baseline in the IBDQ (Inflammatory Bowel Disease Questionnaire) item assessing abdominal bloating by visit in the Full Analysis Set. The analysis excludes patients with the maximum baseline score for the bloating item. Data are presented as mean (SD) by treatment group. * *p*-value from non-parametric test. ** *p*-value from non-parametric test intragroup.

				Change from Baseline				
	N	Mean	SD	N	Mean	SD	*p* Value *	*p* Value **
How much of the time during the last 2 weeks have you been troubled by a feeling of abdominal bloating?								
Placebo								
Baseline	24	3.33	1.81					
Week 4	21	4.24	1.79	21	0.62	1.16		0.1062
Week 8	21	4.14	1.77	20	0.90	1.83		0.1435
Week 10	20	3.95	1.79	20	0.60	1.39		0.2597
VSL#3^®^								
Baseline	26	3.62	1.79					
Week 4	27	4.63	1.71	26	0.96	1.56	0.2552	0.0466
Week 8	24	4.46	1.50	23	1.04	1.72	0.3876	0.0957
Week 10	21	4.81	1.83	20	1.35	1.87	0.1165	0.0296

## Data Availability

The data presented in this study are available on request from the corresponding author.

## Supplementary Materials

The following supporting information can be downloaded at: https://www.mdpi.com/article/10.3390/nu18142257/s1, Table S1: Faecal calprotectin (µg/g) over time and change from baseline (Full Analysis Set); Table S2: IBS Quality of Life (IBS-QoL) total score and individual domain scores by week; Table S3: Mixed model analysis for Inflammatory Bowel Disease Questionnaire (IBDQ) total score.

## Abbreviations

The following abbreviations are used in this manuscript:

UC	Ulcerative colitis
IBS	Inflammatory bowel syndrome
IBS-SSS	Irritable Bowel Syndrome Symptom Severity Scale scores
IBD	Inflammatory bowel disease
IBDQ-32	Inflammatory Bowel Disease Questionnaire
SGA	Subject’s Global Assessment
SCCAI	Simple Clinical Colitis Activity Index
