# Supplementary material for: VSL#3® May Reduce Abdominal Pain and Bloating in Ulcerative Colitis Remission with IBS-like Symptoms: An Exploratory Randomized, Double-Blind Placebo-Controlled Trial
Source: Nutrients. 2026 Jul 10;18(14):2257. doi: 10.3390/nu18142257 (PMC13415462; doi:10.3390/nu18142257)
Supplement: Supplementary file 1 [file nutrients-18-02257-s001.zip › nutrients-4376950-supplementary.pdf]

Supplementary Table S1. Faecal calprotectin (µg/g) over time and change from baseline (Full Analysis Set).

					CHANGE FROM BASELINE			
		N	Mean	SD	N	Mean	SD	pvalue
Faecal Calprotectin (µg/g)								
Placebo								
	Baseline	26	82.18	85.76				
	Week 4	25	78.96	40.66	25	-4.50	85.97	
	Week 8	26	78.22	56.96	26	-3.96	107.72	
VSL#3®								
	Baseline	27	74.83	40.86				
	Week 4	26	96.77	111.02	26	25.60	111.85	0.8954
	Week 8	26	75.12	88.61	26	-0.66	72.08	0.1052

Faecal calprotectin concentrations (µg/g) at baseline, Week 4, and Week 8 by treatment group in the Full Analysis Set (FAS). Data are presented as number of patients (N), mean, and standard deviation (SD). Between-group comparisons of change from baseline at each visit were performed using the non-parametric Mann–Whitney test. P-values refer to between-group comparisons of change from baseline.

Supplementary Table S2. IBS Quality of Life (IBS-QoL) total score and individual domain scores by week.

				CHANGE FROM BASELINE				
	N	Mean	SD	N	Mean	SD	pvalue*	pvalue**
IBS QoL score								
VSL#3®								
Baseline	26	68.47	21.29					
Week 4	27	72.00	19.91	26	2.60	9.14	0.3260	0.4546
Week 8	24	73.71	20.15	23	4.99	11.79	0.3980	0.4257
Week 10	20	71.73	21.36	19	4.72	12.07	0.0421	0.5948
Placebo								
Baseline	25	66.76	20.88					
Week 4	23	69.79	17.58	23	1.57	8.23		0.6130
Week 8	22	70.22	21.35	21	3.61	11.19		0.4363
Week 10	22	65.54	19.35	22	-2.14	12.75		0.7980
Score for Dysphoria								
VSL#3®								
Baseline	26	68.99	25.14					
Week 4	27	71.18	24.30	26	1.44	13.18	0.3583	0.7687
Week 8	24	74.35	23.75	23	4.62	13.29	0.2002	0.3760
Week 10	20	70.00	25.50	19	1.64	14.22	0.3368	0.9204
Placebo								
Baseline	25	67.00	24.62					
Week 4	23	72.83	20.44	23	3.13	10.70		0.4024
Week 8	22	74.29	24.64	21	7.14	12.15		0.2353
Week 10	22	70.03	21.51	22	0.57	14.06		0.7812
Score for Interference With Activity								
VSL#3®								
Baseline	26	61.40	23.23					
Week 4	27	67.86	21.08	26	5.22	10.75	0.0281	0.3774
Week 8	24	68.45	21.91	23	5.90	12.07	0.1391	0.2389
Week 10	20	66.61	24.75	19	4.51	12.65	0.0327	0.4841
Placebo								
Baseline	25	61.43	26.33					
Week 4	23	61.96	23.92	23	-0.16	9.84		0.9753
Week 8	22	61.85	27.50	21	1.36	14.08		0.9319
Week 10	22	56.66	25.09	22	-4.87	16.07		0.4877
Score for Body Image								
VSL#3®								
Baseline	26	71.88	21.81					
Week 4	27	74.77	21.96	26	1.92	13.08	0.4434	0.5598
Week 8	24	75.00	23.09	23	3.80	16.18	0.3516	0.5437
Week 10	20	74.69	21.22	19	3.62	13.55	0.1755	0.6378
Placebo								
Baseline	25	65.75	25.90					
Week 4	23	69.29	22.92	23	2.17	12.59		0.6785
Week 8	22	71.02	24.36	21	5.65	16.29		0.4797
Week 10	22	63.92	24.32	22	-2.56	16.33		0.7318

Score for Health Worry								
VSL#3®								
Baseline	26	65.06	26.14					
Week 4	27	69.75	23.24	26	4.17	13.39	0.1885	0.5072
Week 8	24	70.14	24.07	23	5.07	15.64	0.3173	0.4632
Week 10	20	67.50	23.86	19	8.33	14.96	0.0269	0.8236
Placebo								
Baseline	25	61.67	21.11					
Week 4	23	64.86	20.71	23	1.81	12.30		0.4792
Week 8	22	65.15	24.35	21	3.17	15.92		0.4271
Week 10	22	62.50	24.50	22	-0.38	15.10		0.8635
Score for Food Avoidance								
VSL#3®								
Baseline	26	58.65	21.66					
Week 4	27	62.65	22.69	26	2.56	13.29	0.3502	0.6344
Week 8	24	63.54	24.67	23	4.71	16.05	0.1905	0.4343
Week 10	20	62.08	24.70	19	6.14	18.39	0.0550	0.5919
Placebo								
Baseline	25	54.67	28.37					
Week 4	23	53.26	25.09	23	-0.72	19.45		0.9009
Week 8	22	53.79	26.07	21	1.59	18.93		0.9914
Week 10	22	47.35	29.03	22	-6.06	19.10		0.3978
Score for Social Reaction								
VSL#3®								
Baseline	26	74.28	23.14					
Week 4	27	76.85	21.15	26	1.68	10.83	0.3183	0.7252
Week 8	24	78.91	19.58	23	4.35	15.01	0.2990	0.5623
Week 10	20	80.31	22.24	19	7.24	15.06	0.0526	0.3616
Placebo								
Baseline	25	76.75	19.39					
Week 4	23	79.08	16.17	23	1.09	10.26		0.6928
Week 8	22	78.41	20.75	21	1.49	10.25		0.6271
Week 10	22	74.15	21.15	22	-3.13	14.27		0.7152
Score for Sexual								
VSL#3®								
Baseline	26	77.88	27.68					
Week 4	27	78.24	26.99	26	-0.48	17.13	0.4736	0.9031
Week 8	24	81.77	26.06	23	4.35	14.89	0.1986	0.4471
Week 10	20	80.00	28.21	19	5.26	9.61	0.1329	0.6566
Placebo								
Baseline	25	80.00	29.76					
Week 4	23	85.33	18.71	23	2.72	16.41		0.9206
Week 8	22	85.80	19.01	21	1.79	14.41		0.7366
Week 10	22	82.39	22.38	22	0.57	19.47		0.9174
Score for Relationship								
VSL#3®								
Baseline	26	78.21	24.16					

Week 4	27	81.17	23.64	26	2.24	11.68	0.4338	0.5341
Week 8	24	84.03	21.97	23	6.88	11.42	0.0942	0.2787
Week 10	20	81.25	25.05	19	6.14	14.66	0.0343	0.4647
Placebo								
Baseline	25	75.00	22.44					
Week 4	23	79.35	19.76	23	2.54	10.49		0.5363
Week 8	22	78.03	22.20	21	3.17	13.04		0.6119
Week 10	22	75.00	23.00	22	-0.76	17.99		0.9742

Descriptive statistics for the IBS Quality of Life (IBS-QoL) total score and individual domain scores (Dysphoria, Interference with Activity, Body Image, Health Worry, Food Avoidance, Social Reaction, Sexual, and Relationship) by study visit and treatment group in the Full Analysis Set. Data are presented as mean, standard deviation, and change from baseline, where applicable. * p-value from non-parametric test. ** p-value from non-parametric test intra-group.

Supplementary Table S3. Mixed model analysis for Inflammatory Bowel Disease Questionnaire (IBDQ) total score

			Change from baseline				Difference in reduction		
	VSL#3®	Placebo	VSL#3®	p value	Placebo	p value	Mean(se)	CI 95%	p value
Baseline	158.4 (3.22)	158.6 (3.29)							
Week 4	164.6 (3.22)	160.6 (3.46)	6.23 (3.51)	0.0785	1.99 (3.74)	0.5957	-4.24 (5.13)	(-14.4 ; 5.92)	0.4104
Week 8	167.3 (3.38)	167.1 (3.52)	8.89 (3.66)	0.0165	8.49 (3.80)	0.0272	-0.40 (5.28)	(-10.8 ; 10.04)	0.9400
Week 10	171.2 (3.56)	164.1 (3.52)	12.81 (3.83)	0.0011	5.41 (3.80)	0.1571	-7.40 (5.40)	(-18.1 ; 3.27)	0.1724

Results of the mixed-effects model evaluating changes from baseline in the Inflammatory Bowel Disease Questionnaire (IBDQ) total score over time in the Full Analysis Set. The model includes patient and study center as random effects and baseline value, visit, treatment group, and treatment-by-visit interaction as fixed effects.
